# Beyond Smoking: Emerging Drivers of COPD and Their Clinical Implications in Low- and Middle-Income Countries: A Narrative Review

**DOI:** 10.3390/jcm14134633

**Published:** 2025-06-30

**Authors:** Ramona Cioboata, Mara Amalia Balteanu, Denisa Maria Mitroi, Sidonia Catalina Vrabie, Silviu Gabriel Vlasceanu, Gabriela Marina Andrei, Anca Lelia Riza, Ioana Streata, Ovidiu Mircea Zlatian, Mihai Olteanu

**Affiliations:** 1 Department of Pneumology, University of Medicine and Pharmacy, 200349 Craiova, Romania; ramona_cioboata@yahoo.com (R.C.); gabriela.andrei@umfcv.ro (G.M.A.); mihai.olteanu@umfcv.ro (M.O.); 2 Department of Pneumology, Victor Babes University Hospital, 200515 Craiova, Romania; 3 Department of Pulmonology, Faculty of Medicine, Titu Maiorescu University, 031593 Bucharest, Romania; mara.balteanu@prof.utm.ro; 4 Doctoral School, University of Medicine and Pharmacy, 200349 Craiova, Romania; denisa_maria2@yahoo.com; 5 Department of Obstetrics and Gynecology, University of Medicine and Pharmacy of Craiova, 200349 Craiova, Romania; 6 Department of Microbiology, “Carol Davila” University of Medicine and Pharmacy, 050474 Bucharest, Romania; 7 Department of Thoracic Surgery, Marius Nasta Pneumology Institute, 050159 Bucharest, Romania; 8 Regional Centre of Medical Genetics Dolj, Emergency County Hospital Craiova, 200642 Craiova, Romania; anca_riza@yahoo.com (A.L.R.); ioana.streata@umfcv.ro (I.S.); 9 Laboratory of Human Genomics, University of Medicine and Pharmacy of Craiova, 200638 Craiova, Romania; 10 Department of Microbiology, University of Medicine and Pharmacy of Craiova, 200349 Craiova, Romania; ovidiu.zlatian@umfcv.ro

**Keywords:** chronic obstructive pulmonary disease, COPD, low- and middle-income countries, LMICs, non-smoking risk factors, COPD phenotypes

## Abstract

Chronic obstructive pulmonary disease (COPD) is an escalating global health burden, with a disproportionate impact on low- and middle-income countries (LMICs). Although tobacco smoking is a well-established risk factor, emerging evidence highlights the significant role of non-smoking exposure in driving the prevalence of COPD in these regions. This narrative review synthesizes current data on key non-smoking contributors, including household air pollution, ambient urban pollution, occupational exposure, early-life respiratory insults, chronic infections, and socioeconomic adversity. These risk factors are associated with distinct COPD phenotypes, often marked by increased airway inflammation, reduced emphysema, and variable airflow limitation. Such presentations are particularly common among women and younger populations in LMICs. However, diagnostic and therapeutic challenges persist, owing to limited disease awareness, under-resourced health systems, restricted access to essential medications, and financial constraints impacting adherence. Despite the proven effectiveness of non-pharmacological measures and public health interventions, their implementation remains inadequate because of infrastructural and funding limitations. Bridging these gaps requires region-specific clinical guidelines, improved diagnostic infrastructure, expanded access to affordable treatment, and culturally sensitive interventions. Future priorities include identifying robust biomarkers, refining disease definitions to accommodate non-smoking phenotypes, and advancing implementation science to improve interventions. A coordinated, context-aware global response is essential to reduce the growing burden of COPD in LMICs and to ensure equitable respiratory health outcomes.

## 1. Introduction

Chronic obstructive pulmonary disease (COPD) is the third leading cause of death worldwide, with nearly 90% of its fatalities and over 80% of its 315.5 million cases occurring in low- and middle-income countries (LMICs) as of 2019, where prevalence ranges from 4.1% to 22.2% across regions [1,2]. Between 2007 and 2017, deaths due to COPD increased by 17.5%, reaching approximately 3.2 million in 2017 [3]. In 2018, around 12.8 million adults, representing 5% of the adult population, were diagnosed with COPD [4,5].

Updated global data on chronic respiratory diseases (CRDs) are vital for achieving the third UN Sustainable Development Goal of reducing premature mortality from non-communicable diseases by 2030. CRDs caused approximately 4.0 million deaths globally in 2019, ranking as the third leading cause of death, with a prevalence of 454.6 million cases, a 39.8% increase since 1990. COPD contributed most significantly, with 3.3 million deaths among 212.3 million cases, whereas asthma had the highest prevalence, with 262.4 million cases. Notably, interstitial lung disease and pulmonary sarcoidosis have increased, despite a reduction in the incidence of other CRDs. Smoking, air pollution, and occupational exposure remain major global risk factors, particularly in LMICs, with the highest mortality and DALY rates [6].

Globally, COPD prevalence is estimated to be 10.3% [1,7], and cases among adults over 25 years old are projected to increase by approximately 23% by 2050, disproportionately affecting women and LMIC populations [8]. Rising smoking rates in LMICs, aging populations in high-income regions, and high biomass exposure will likely drive further prevalence increases, including among non-smokers [9]. Currently, up to 95% of COPD cases in LMICs remain undiagnosed because of limited access to spirometry, medication, and pulmonary rehabilitation [10,11]. The high prevalence of tobacco, biomass fuel smoke, ambient air pollution, occupational hazards, and malnutrition in these regions exacerbates vulnerability, compounded by demographic factors, such as male sex and low BMI. Limited availability of respiratory specialists and low awareness among healthcare providers further impede diagnosis and management [10,12]. Addressing this burden requires targeted education, cost-effective screening tools, reduced risk exposure, improved diagnostic and therapeutic access, and integration of COPD management into national programs [11].

Although tobacco remains the primary cause of COPD, approximately 25–45% of cases occur in non-smokers, often due to biomass smoke exposure, which affects around 3 billion people globally, far exceeding the number of tobacco users [13,14,15]. Biomass smoke from cooking and heating significantly impacts women and children in LMICs, while occupational exposures and outdoor air pollution further increase the risk [15].

The global increases in electronic cigarette (e-cigarette) use raise concerns regarding COPD risks. Studies have reported an elevated COPD risk among current users (OR 1.44–1.75), particularly daily users, and higher risks among dual users than among single-product users. A dose–response relationship links increased e-cigarette use to heightened respiratory symptoms and an elevated risk of COPD through mechanisms such as oxidative stress and inflammation [16,17,18]. Evidence on e-cigarettes as a means of smoking cessation or as harm reduction tools in COPD remains limited and inconclusive due to methodological constraints [19,20].

Emerging evidence emphasizes COPD’s independent development from smoking due to diverse environmental, occupational, and genetic risk factors. Early-life exposures, including maternal smoking, low birth weight, prematurity, and childhood respiratory infections, significantly influence COPD risk later in life. Serious respiratory infections and asthma during childhood substantially elevate COPD risk (aOR 2.23–3.45), underscoring the importance of early prevention strategies [21]. Respiratory infections, genetic predispositions such as alpha-1 antitrypsin deficiency, and socioeconomic disadvantages further compound COPD susceptibility [15,22].

This narrative review synthesizes current evidence on non-smoking drivers of COPD in LMICs, focusing on household biomass smoke, air pollution, occupational exposures, respiratory infections, genetic factors, and socioeconomic determinants, emphasizing implications for the diagnosis, management, and prevention in resource-constrained settings.

## 2. Emerging Non-Smoking Drivers

### 2.1. Pollution-Related Factors

**Figure 1:**
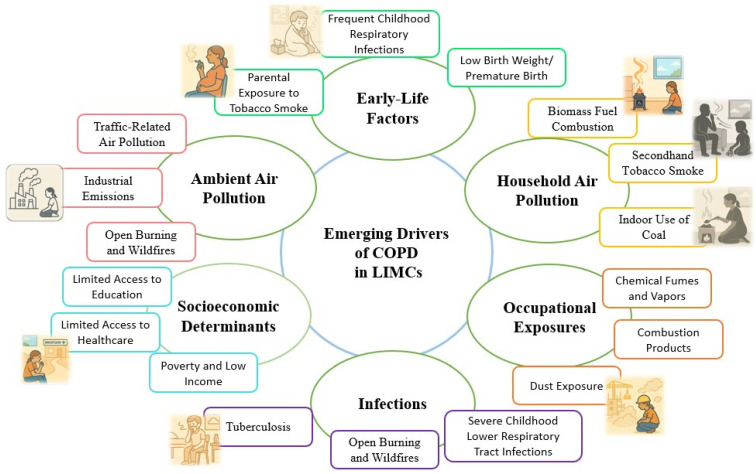
Emerging non-smoking drivers of chronic obstructive pulmonary disease (COPD) in low- and middle-income countries (LMICs).

Pollution-related factors, encompassing both household and ambient air pollution, have emerged as critical contributors to the development and progression of COPD, particularly within LMICs (Figure 1).

Household air pollution (HAP), primarily from indoor combustion of solid fuels (wood, coal, biomass) for cooking and heating, significantly impacts global respiratory health, particularly affecting women and children. Similarly, ambient air pollution—exposure to particulate matter (PM_2.5_, PM_10_), nitrogen dioxide (NO_2_), and ozone (O_3_)—is increasingly recognized as a major COPD risk factor in urban areas [23,24]. Both pollution types induce chronic respiratory inflammation, underscoring the need for targeted mitigation strategies and policy interventions.

COPD ranks prominently among respiratory diseases associated with HAP, disproportionately affecting vulnerable populations such as women and children in resource-poor settings [23,24]. Epidemiological studies indicate that individuals exposed to HAP have approximately a 41% higher likelihood of developing COPD, with the risk particularly pronounced among women who regularly engage in cooking and other domestic activities in environments with high indoor pollutant levels [24,25]. In specific populations, HAP exposure alone may account for up to 13.5% of COPD cases, a proportion comparable to that attributed to cigarette smoking [23]. High-altitude rural communities, characterized by severe resource constraints, often experience elevated indoor particulate matter (PM_2.5_) concentrations and consequently a higher COPD prevalence [24].

Pathophysiologically, household air pollution (HAP) contributes to COPD via persistent airway inflammation, reduced lung function, and fibrotic airway changes, notably with less emphysema compared to tobacco-related COPD [26,27]. Clinically, greater HAP exposure is associated with chronic cough, phlegm, wheezing, spirometric decline, and elevated exhaled carbon monoxide [26]. Women and children experience heightened morbidity due to prolonged indoor exposure, highlighting significant gender and age disparities in COPD risk [25].

Research on HAP and COPD faces methodological challenges, primarily due to reliance on indirect exposure proxies and subjective outcomes rather than objective pollution measures, complicating causality assessments [28]. Confounding factors such as poverty, tobacco use, and ambient air pollution further complicate attribution solely to HAP, underscoring the need for high-quality studies with standardized diagnostics and precise pollutant monitoring [28].

Global trends show a declining COPD burden from HAP (1990–2019), while ambient particulate pollution-linked COPD is increasing. In regions with low socio-demographic indices (SDIs), HAP remains a greater contributor than ambient pollution. Thus, interventions promoting improved cookstoves, cleaner fuels, and clean energy adoption are crucial [25,27].

HAP contributes to COPD through airway inflammation, oxidative stress, epithelial barrier dysfunction, impaired immunity, and genetic susceptibility [26,27,29]. Chronic pollutant inhalation triggers sustained inflammation, airway remodeling, and airflow limitation. Epithelial damage increases infection susceptibility, exacerbating inflammation and lung injury. Impaired macrophage function further predisposes individuals to recurrent infections, worsening COPD progression [27,30]. Genetic and epigenetic factors also modulate susceptibility [29]. Rural high-altitude communities relying on biomass fuels exhibit distinctively higher COPD prevalence, primarily with airway disease rather than emphysema [24]. Addressing these mechanisms through targeted interventions is vital for reducing COPD burden.

Secondhand smoke (SHS) exposure significantly influences pulmonary hypertension (PH) development, a serious condition frequently linked to COPD. SHS exposure correlates with increased COPD risk, demonstrating a dose–response relationship [31,32]. Infants exposed to parental smoking show elevated pulmonary arterial hypertension (PAH) incidence, directly proportional to parental smoking intensity [33]. Mechanistically, SHS induces endothelial dysfunction, oxidative stress, and vascular remodeling, increasing pulmonary artery pressures [34]. Mitigating SHS exposure is thus essential for reducing PH incidence.

HAP remains a preventable cause of COPD, particularly in LMICs. Improved exposure assessment, control of confounders, and investments in clean cooking technologies are crucial. Concurrently, ambient air pollution significantly impacts the risk of COPD, particularly in urban settings. Exposure to PM_2.5_, PM_10_, NO_2_, O_3_, SO_2_, and CO disproportionately affects older adults, women, and lower socioeconomic groups, exacerbating COPD incidence, progression, and morbidity [35,36,37,38].

Robust epidemiological evidence links long-term PM_2.5_ and NO_2_ exposure with increased COPD incidence and lung function decline, with even modest increases in exposure significantly elevating risk (HR up to 1.18 and 1.07 per 10 μg/m^3^, respectively) [35,37,39]. Short-term pollution spikes acutely increase COPD exacerbation risks and reduce lung function parameters (e.g., FEV1) [38]. Each 10 μg/m^3^ increase in PM_2.5_ is associated with a 3.1% increase in COPD hospital admissions, a 2.5% increase in COPD-related mortality, and an 18% increase in long-term COPD incidence [38,40,41]. Socioeconomically disadvantaged groups experience amplified adverse respiratory effects due to higher baseline exposures and compounded vulnerabilities, emphasizing targeted public health strategies [35,37].

Urbanization independently increases COPD risk, while residential greenness offers protective benefits [37]. High-density neighborhoods reduce physical activity among Patients with COPD, whereas pedestrian-friendly areas promote better respiratory outcomes [42].

In conclusion, reducing exposure to ambient urban air pollutants remains essential not only for COPD prevention but also for protecting vulnerable populations who disproportionately suffer from this condition. Alongside household air pollution, ambient air pollutants necessitate comprehensive and coordinated strategies aimed at minimizing exposure, mitigating adverse respiratory outcomes, and ensuring equitable respiratory health, particularly within vulnerable and underserved communities.

### 2.2. Occupational Exposures

Occupational exposure significantly contributes to the COPD burden, particularly in LMICs, yet remains frequently underrecognized. Industries such as mining, construction, agriculture, metal and foundry work, textile production, automotive repair, and informal jobs involving biomass smoke notably affect lung health, especially among women in rural areas [43,44,45,46].

Occupational pollutants exacerbate COPD symptoms, causing increased airflow limitation, frequent exacerbations, and higher rates of hospitalization [44,47,48,49]. Common symptoms include chronic sputum production, dyspnea, and reduced quality of life. Notably, the combined exposure to tobacco smoke and occupational pollutants synergistically elevates COPD risk beyond individual effects [43].

Occupational exposure accounts for approximately 10–21% of COPD cases globally, with higher prevalence in specific occupations and regions [43,45,50,51,52]. Harmful exposures include mineral and biological dust, diesel exhaust, welding fumes, pesticides, and organic particles. Agricultural and textile workers exposed to biological dust show relative risks (RRs) between 1.33 and 1.6; miners and construction workers exposed to silica and mineral dust have hazard ratios (HRs) ranging from 1.46 to 1.71; welding fumes and diesel exhaust present additional HRs of 1.57 and 1.18, respectively. Agricultural pesticide exposure carries a particularly high risk (RR 2.2), and biomass smoke exposure among women in LMICs has an odds ratio (OR) above 1.5 [43,44,45,46,51]. The FLOW study, which examined 1202 patients with COPD, reported an increased COPD risk associated with exposure to vapors, gas, dust, or fumes (VGDF) (OR 2.11; 95% CI 1.59–2.82; PAF 31%). Using a job exposure matrix (JEM), they found an OR of 2.27 (95% CI 1.46–3.52; PAF 13%) [43].

The co-occurrence of occupational exposures and smoking substantially heightens COPD risk. The FLOW study demonstrated a synergistic interaction, with an OR of 14.1 (95% CI 9.33–21.2) for COPD development, significantly exceeding risks from either exposure alone. This results in earlier disease onset, more severe airflow obstruction, worsened symptoms, and increased hospitalizations [43].

Addressing COPD in LMICs requires integrated prevention strategies, combining occupational safety enforcement and smoking cessation programs, to effectively mitigate the compounded risk from dual exposures [44,47,48].

### 2.3. Socioeconomic and Behavioral Determinants

Socioeconomic deprivation significantly influences COPD outcomes. A systematic review of 76 studies consistently linked low socioeconomic status (SES) to poorer quality of life and reduced healthcare access in patients with COPD. These findings emphasize the need to investigate underlying factors, including patient beliefs and behaviors, currently explored in ongoing research [53].

Socioeconomic and behavioral factors, along with healthcare access, significantly impact COPD development, progression, and outcomes. Individuals with lower SES experience higher rates of COPD, worse health outcomes, and more barriers to effective care.

Along with age and sex, socioeconomic status is one of the most powerful determinants of health. A systematic review examining the association between socioeconomic status and COPD health outcomes demonstrated a consistent and significant inverse relationship [54]. Across diverse populations and SES measures, nearly all studies found that individuals in the lowest socioeconomic strata were at least twice as likely to have poor COPD outcomes compared to those in the highest socioeconomic groups, with the range in some studies spanning up to a ten-fold difference. These findings underscore that social and economic disadvantage exerts a major, consistent impact on both COPD morbidity and mortality, reinforcing the urgent need for public health strategies and research to address SES disparities in COPD populations [54].

Lower educational attainment and household income strongly correlate with higher COPD prevalence, greater disease severity, and poorer health-related quality of life (HRQoL) [53,55,56]. Individuals from lower SES groups often face occupational exposures to inhalant toxins and indoor biomass fuels, increasing their COPD risk [54,55,57,58,59]. A composite SES measure including education, income, and household size further highlights the link between lower SES and increased COPD prevalence.

Tobacco use is more prevalent among lower SES populations, significantly increasing COPD risk and severity [54]. Other behavioral factors, such as poor housing, inadequate nutrition, and higher infection rates also contribute to COPD progression [60].

Lower SES individuals encounter barriers to healthcare access, including medication affordability, limited access to diagnostic services such as spirometry, and reduced participation in pulmonary rehabilitation. Geographic and systemic factors, such as rural residence and under-resourced health systems, exacerbate these barriers and result in higher hospitalization rates, more frequent exacerbations, and increased mortality among patients with COPD [56,61].

Effective interventions include enhanced surveillance, education for patients and healthcare providers, affordable care access, smoking cessation programs, and occupational exposure reduction. Public health policies targeting SES disparities are essential for improving COPD outcomes among disadvantaged populations [53,57].

### 2.4. Early-Life Factors

Early-life factors significantly influence the development of COPD, as events and exposures during prenatal and early childhood periods profoundly affect lung growth and function. Factors such as maternal smoking, parental asthma, low birth weight, prematurity, and early-life respiratory infections can predispose individuals to COPD later in life, underscoring the critical importance of adequate nutrition during fetal life and early childhood to ensure optimal lung development and reduce lifelong respiratory vulnerability [62,63,64]. In a meta-analysis of 30 studies including 795,935 participants, serious childhood respiratory infections, such as pneumonia or bronchitis, were associated with more than a two-fold increase in the risk of COPD (adjusted odds ratio [aOR] 2.23, 95% CI 1.63–3.07), while childhood asthma increased the risk by over three-fold (aOR 3.45, 95% CI 2.37–5.02). Additional risk factors included maternal smoking (aOR 1.42, 95% CI 1.17–1.72), child maltreatment (aOR 1.30, 95% CI 1.18–1.42), and low birth weight (aOR 1.58, 95% CI 1.08–2.32). These data underscore the importance of early-life prevention strategies to markedly reduce the long-term risk of COPD [21].

Poor nutrition and growth deficits, manifested through low birth weight and prematurity, result in impaired lung maturation and decreased lung function, which extend into adulthood and significantly increase COPD susceptibility. Low birth weight alone is associated with a 1.58-fold increased risk of COPD [21]. Interventions aimed at improving maternal nutrition, prenatal care, and childhood nutritional status could substantially mitigate these risks, underscoring the importance of nutritional support and growth monitoring from pregnancy through early childhood. Early-life adverse events, such as respiratory infections and nutritional deficits, interact with genetic predispositions to permanently alter lung development trajectories. These changes result in persistently reduced lung function, increased susceptibility to respiratory illnesses, and a heightened lifetime risk of COPD. Prevention efforts targeted at early-life exposures including reducing maternal smoking, enhancing antenatal care, ensuring adequate nutrition, promoting timely immunization, and reducing pollutant exposures represent critical strategies to decrease the future burden of COPD [64,65].

### 2.5. Infection-Related Factors

Childhood respiratory infections, such as pneumonia, bronchitis, and severe bronchiolitis, significantly elevate COPD risk by impairing lung development and inducing chronic inflammation, resulting in lasting structural lung damage and reduced lung function into adulthood. Multiple severe early-life infections increase COPD risk by up to 2.23 times, emphasizing the importance of immunization and the effective management of childhood respiratory diseases [64,65,66]. Effective immunization strategies and improved management of childhood respiratory illnesses are essential interventions for reducing this long-term risk. Furthermore, exposure to maternal smoking enhances the risk, particularly when combined with low birth weight [21].

Throughout life, recurrent respiratory infections, both viral (rhinovirus, RSV, influenza, adenovirus) and bacterial (*Streptococcus pneumoniae*, *Haemophilus influenzae*, *Moraxella catarrhalis*), substantially contribute to COPD development, exacerbations, accelerated lung function decline, and increased mortality. Chronic infections with Chlamydia pneumoniae and Helicobacter pylori also independently raise COPD risk [67,68].

The COVID-19 pandemic introduced additional complications, with persistent respiratory symptoms and organ dysfunction (post-COVID-19 syndrome) potentially accelerating COPD progression [69,70]. The severity and prevalence of this syndrome are associated with initial disease severity and underlying comorbidities, highlighting the need for integrated post-viral care [71,72].

Tuberculosis (TB) remains a significant public health issue, especially in LMICs, with rising incidence exacerbated by pandemic-related diagnostic delays [73]. History of TB markedly increases COPD risk, severity, symptom frequency, exacerbations, and hospitalizations [73,74]. Patients with prior pulmonary TB exhibit significantly reduced lung function (lower FEV1% and FEV1/FVC) and greater COPD severity, emphasizing the necessity of integrated TB and COPD management [75].

TB and COPD share chronic inflammatory mechanisms involving immune cell activation, cytokine overproduction (IL-1β, IL-6, TNF-α, IFN-γ), and impaired tissue repair, contributing to airway remodeling and persistent airflow limitation. This overlap creates a distinct clinical entity, “tuberculosis-associated obstructive pulmonary disease” (TOPD), characterized by severe impairment and exacerbation risk [76]. Chronic inflammation from COPD may weaken immune defenses, facilitating TB persistence or reactivation, while TB-induced lung damage predisposes patients to COPD [77].

Conversely, COPD significantly elevates active TB infection risk, making it up to three times higher than in the general population, and increases susceptibility to other respiratory infections, particularly in immunocompromised individuals [78,79]. Shared risk factors such as smoking, pollution, malnutrition, and poverty, alongside genetic predispositions and immune dysregulation, further link TB and COPD. Clinical COPD management, notably inhaled corticosteroid use, can inadvertently heighten TB risk, particularly in high TB prevalence areas [80,81].

This bidirectional relationship underscores the importance of comprehensive prevention strategies, including early TB detection, prompt and effective TB treatment, and cautious COPD management, especially regarding corticosteroid use. Addressing these intertwined infections through integrated clinical and public health approaches is crucial to reducing their combined global burden.

### 2.6. Genetic and Epigenetic Determinants

Genetic and epigenetic factors significantly influence COPD susceptibility and pathogenesis among non-smokers, extending our understanding beyond tobacco-related risks. Several genetic variants have been associated with non-smoking COPD, including single nucleotide polymorphisms in ADAM33, IL-1RN, IL-1B, IL-6, TNF-α, SOD2, CAT, and AQP5, with certain ADAM33 variants specifically linked to non-smokers, indicating distinct risk profiles [82]. Genome-wide analyses highlight shared genetic loci for airflow obstruction between smokers and non-smokers, yet also identify novel loci, such as KANSL1, TSEN54, TET2, and RBM19/TBX5, which are uniquely relevant for lung function irrespective of smoking history [83].

Epigenetic studies in never-smokers have identified unique DNA methylation sites associated with lung function (FEV1/FVC), including previously unrecognized CpG sites, emphasizing the role of epigenetic regulation in COPD risk [83]. Accelerated epigenetic aging and differential methylation in epithelial biology and cellular adhesion genes correlate with COPD progression independent of smoking. Altered gene expression impacting mitochondrial function, cellular senescence, and telomere maintenance further implicates aging and cellular stress pathways in disease pathogenesis [84,85]. Epigenetic changes, including DNA methylation and histone acetylation, also modulate critical inflammatory pathways (e.g., TGF-β1, CXCL8) independently of tobacco exposure.

These genetic and epigenetic insights highlight potential biomarkers for COPD risk and progression, supporting personalized diagnostic and therapeutic strategies and emphasizing the need to integrate non-smoking-related mechanisms into research and clinical practice [82,86].

## 3. Clinical Implications

### 3.1. Phenotypic Variability

COPD is characterized by significant phenotypic variability, reflecting differences in clinical presentations, disease progression, and treatment responses among patients. Phenotypic variations extend beyond traditional categories of chronic bronchitis and emphysema, encompassing additional subtypes such as the asthma–COPD overlap syndrome (ACOS), the frequent exacerbator phenotype (COPD-D), the rapid-decline phenotype, and the predominant small-airway disease phenotype [87].

The chronic bronchitis phenotype is characterized by chronic cough and sputum production and is linked to higher exacerbation rates, frequent infections, and increased healthcare utilization. The emphysema phenotype is identified by substantial lung parenchymal destruction. Patients typically present with severe dyspnea, significant airflow obstruction, and reduced diffusion capacity. This phenotype is associated with rapid functional decline and higher mortality [87]. The asthma–COPD overlap (ACOS) presents with features of both asthma and COPD, including variable airflow limitation, greater reversibility, increased airway hyperresponsiveness, and enhanced response to inhaled corticosteroids, with ACOS patients generally experiencing frequent exacerbations and poorer outcomes compared to other phenotypes [88].

COPD-D, defined as experiencing two or more moderate to severe exacerbations per year, typically presents with more severe symptoms, greater airflow limitation, and worse health-related quality of life compared to infrequent exacerbators (COPD-I), which are characterized by one or no exacerbations annually [89,90,91]. Biological differences underpinning these phenotypes include distinct proteomic and immune profiles, with COPD-D patients exhibiting altered immune responses (e.g., increased IgA production and phenylalanine metabolism) and persistent inflammation [92]. COPD-D is also associated with advanced imaging abnormalities, such as higher emphysema indices and pronounced airway wall thickening, predictive of further exacerbations and hospitalizations [93,94]. Key clinical predictors distinguishing COPD-D from COPD-I include older age, lower FEV1, higher eosinophil counts, history of severe exacerbations, smoking, and greater imaging evidence of emphysema [90,93,94]. Given the significantly poorer prognosis of frequent exacerbators, characterized by accelerated lung function decline, increased hospital readmissions, and higher mortality, accurate phenotype identification is crucial for optimizing clinical management and improving patient outcomes [90].

The rapid-decline phenotype is characterized by accelerated reduction in lung function, with this subgroup showing a steeper trajectory of disease progression and highlighting the need for early identification and aggressive management. Identifying and understanding these phenotypic differences are essential for personalized treatment strategies, allowing clinicians to optimize management plans, enhance patient outcomes, and improve prognostication [95,96,97].

### 3.2. Non-Smoking Phenotypes

COPD in non-smokers represents a distinct subgroup, influenced by factors such as biomass fuel exposure, occupational exposures, genetic predisposition, and chronic respiratory infections. Non-smoking COPD (NS-COPD) patients frequently exhibit different clinical profiles, with more pronounced airway inflammation, chronic bronchitis symptoms, and less emphysematous lung destruction compared to smoking-related COPD (S-COPD) [95]. The recognition of non-smoking COPD phenotypes is important, as these patients may respond differently to standard COPD treatments, highlighting the importance of targeted therapeutic and preventive strategies tailored to these specific etiologies. Identifying and understanding these phenotypic differences are essential for personalized treatment strategies, allowing clinicians to optimize management plans, enhance patient outcomes, and improve prognostication [87,98,99].

NS-COPD is increasingly recognized as a distinct clinical phenotype, particularly prevalent in regions with significant exposure to non-tobacco risk factors. Key differences between NS-COPD and S-COPD have been observed across clinical, physiological, and structural features. NS-COPD typically presents with small-airway disease, air trapping, and less emphysema, whereas S-COPD is characterized by a greater degree of emphysema and significant reductions in lung diffusion capacity. NS-COPD patients also show a higher prevalence of COPD–bronchiectasis overlap, while emphysema predominates in S-COPD cases. Notably, among non-smokers, females more frequently exhibit an airway-predominant phenotype, characterized by increased airway wall area and less emphysema, whereas males are more likely to have emphysema-predominant disease [97,100].

Research in Mexican women has highlighted key differences between COPD phenotypes arising from biomass smoke exposure versus tobacco smoke. In a cross-sectional study [101], female never-smokers with COPD and biomass exposure exhibited significantly less emphysema but more air trapping than female ex-smokers with tobacco-related COPD, supporting the concept of an airway-predominant phenotype in non-smoking COPD. Radiological assessments demonstrated lower emphysema scores and a smaller extent of emphysematous spaces in the biomass group, while air trapping was notably higher compared to the tobacco group. Furthermore, women with biomass-associated COPD reported worse quality of life in certain domains and had lower oxygen saturation at rest and during exercise. These findings emphasize that non-tobacco exposures such as biomass fuel, environmental tobacco smoke (ETS), and childhood respiratory infections are important risk factors for NS-COPD, particularly among women and in low- and middle-income countries [95,96,98]. This growing body of evidence underlines the necessity for phenotype-specific recognition and targeted management in the non-smoking COPD population. Demographically, NS-COPD patients are often younger, have a higher body mass index (BMI), and display a more balanced gender ratio compared to S-COPD, which predominantly affects males [87,95]. Emerging data from low- and middle-income countries reinforce the notion that NS-COPD is a distinct clinical entity with characteristic phenotypic features. A recent study from India demonstrated that NS-COPD patients tend to present at a younger age and more frequently exhibit the COPD-bronchiectasis overlap phenotype, while smoking-related COPD (S-COPD) patients more commonly display emphysema. NS-COPD is also associated with less severe airflow obstruction, higher rates of postbronchodilator reversibility, and a generally slower rate of lung function decline compared to S-COPD [96]. Although the symptom burden and quality of life are broadly similar between groups, NS-COPD patients may experience slightly better symptom scores and less severe airflow impairment. These findings highlight the importance of recognizing and understanding the distinct phenotypic characteristics of NS-COPD for accurate diagnosis, prognostication, and personalized management in non-smoking populations [97,100,102].

Inflammatory and cellular differences further distinguish NS-COPD. These patients demonstrate lower levels of neutrophilic inflammation and higher eosinophilic presence in sputum relative to S-COPD. In addition, distinct differences in the alveolar macrophage surface markers between NS-COPD and S-COPD suggest different immune response mechanisms [102].

Recognizing the unique characteristics of NS-COPD has significant implications for clinical practice. Diagnostic criteria and therapeutic approaches developed for S-COPD may require adaptation to effectively manage NS-COPD patients. Tailored interventions, including targeted exposure reduction strategies, specific pharmacological treatments, and personalized rehabilitation programs, are essential to address the distinct pathophysiological mechanisms underlying NS-COPD. Continued research is needed to identify precise biomarkers that differentiate NS-COPD from S-COPD and to explore genetic and epigenetic factors in susceptibility and disease progression. Improved understanding and clear differentiation of NS-COPD will facilitate more accurate diagnosis, personalized therapy, and ultimately, better patient outcomes [95,96,101].

## 4. Diagnostic Challenges

**Figure 2:**
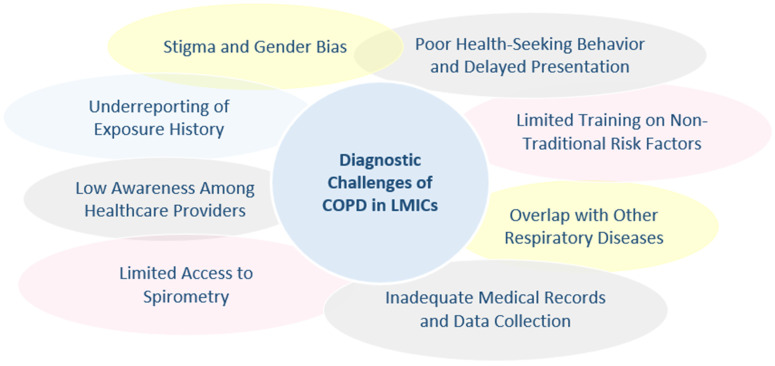
Diagnostic challenges of chronic obstructive pulmonary disease (COPD) in low- and middle-income countries (LMICs).

Diagnosing COPD in non-smokers presents a complex challenge, particularly in LMICs, where non-traditional risk factors such as biomass fuel exposure, prior pulmonary infections, and environmental pollutants are more prevalent. The clinical presentation of NS-COPD often differs markedly from the classic S-COPD phenotype, with patients typically presenting at a younger age, showing less severe airflow obstruction, more frequent bronchiectasis overlap, and reduced emphysema on imaging [96]. These atypical features pose challenges for diagnosis, as standard criteria largely developed for tobacco-related disease tend to have lower sensitivity for NS-COPD, and the symptom profile, often limited to chronic cough and mild airflow limitation, can overlap with other respiratory diseases, increasing the risk of misdiagnosis or underdiagnosis [87]. Moreover, comparative studies highlight that biomass-smoke-induced COPD (BSCOPD), a major cause of NS-COPD in LIMCs, is distinct from tobacco-smoke-induced COPD (TSCOPD) not only in clinical presentation but also in pathogenesis. BSCOPD typically presents with less emphysema and small-airway damage, along with a higher prevalence of pulmonary hypertension, and is further characterized by bronchial hyperresponsiveness, significant hypoxemia, and an inflammatory profile with marked mucous hypersecretion and airway remodeling. In contrast, TSCOPD is associated with more severe airflow obstruction and emphysema. Unique genetic, epigenetic, and oxidative stress mechanisms in BSCOPD add further complexity to diagnosis and management, often leading to underrecognition of the impact of biomass smoke on lung aging and exacerbation risk. These distinctions underscore the urgent need for targeted research to refine diagnostic criteria, identify specific biomarkers, and improve clinical outcomes for populations exposed to biomass smoke [103] (Figure 2).

A significant barrier is the limited awareness among healthcare providers in LMICs regarding the impact of non-smoking risk factors, such as biomass smoke exposure and post-TB airway damage, which remain underrecognized contributors to COPD prevalence [12]. This lack of awareness is compounded by systemic and resource limitations, notably the severely restricted access to essential diagnostic tools such as pulmonary function tests, particularly spirometry. In many low- and middle-income countries (LMICs), spirometry and other advanced pulmonary function assessments are frequently unavailable or prohibitively expensive, significantly hindering timely diagnosis. This diagnostic gap directly contributes to alarmingly high rates of undiagnosed COPD cases, with estimates suggesting that up to 95% of COPD cases remain undiagnosed in certain regions. Recognizing this challenge, international respiratory organizations have recommended that patient and professional groups actively engage policymakers to prioritize and establish accessible lung function testing programs in LMICs. This policy advocacy is critical, emphasizing that effective COPD management relies heavily on the availability and affordability of diagnostic tools integral to guideline-based respiratory care [9,12].

Diagnosing COPD in non-smokers poses unique challenges. Since the disease is often not suspected in lifelong non-smokers, particularly women, the elderly, or those exposed to risk factors such as biomass smoke or occupational pollutants, diagnosis is frequently delayed or missed. Furthermore, overlapping symptoms with other chronic respiratory or cardiac conditions can complicate recognition and lead to underdiagnosis [104].

Non-smoking COPD patients represent a distinct subgroup with a different risk profile for exacerbations compared to their smoking counterparts. Generally, non-smokers with COPD experience a significantly lower risk of recurrent acute exacerbations, exhibit milder lung function impairment, and have reduced mortality rates compared to current or former smokers with COPD [105,106]. These individuals are often older, have a higher body mass index, and display less severe respiratory symptoms and dyspnea scores. Non-smoking COPD is also associated with a lower prevalence of emphysema and lung cancer compared to smoking-related disease [106]. Despite these generally favorable outcomes, certain clinical and biological factors, such as higher symptom burden (elevated COPD Assessment Test scores), increased dyspnea, elevated plasma levels of vascular endothelial growth factor and C-reactive protein, urban residence, and the presence of emphysema remain important predictors of exacerbation risk even among non-smokers. Prognostically, non-smoking COPD patients have lower mortality and severe exacerbation rates, with non-exacerbating individuals more likely to succumb to malignancies or cardiovascular disease than to respiratory causes [107]. Thus, while non-smoking COPD patients generally fare better, careful assessment and management of symptom burden, biomarkers, and comorbidities are essential to further reduce exacerbation risk in this group [104].

Early diagnosis and screening are critical in addressing the high burden of COPD in LMICs, where most cases remain undetected and untreated. A major barrier to effective COPD management in LMICs lies in the significant gap between evidence-based guidelines and real-world practice. Most cases of COPD in LMICs remain undiagnosed, and even among those identified through population screening, the majority receive little or no therapy. Studies from Nepal, Peru, and Uganda show that 95% of individuals with COPD detected by screening had never been previously diagnosed, highlighting a major missed opportunity for intervention. These screening-detected patients also had substantial unmet needs, particularly for non-pharmacological interventions such as education, vaccinations, pulmonary rehabilitation, smoking cessation support, and advice on reducing biomass smoke exposure. Access to pharmacological therapies was also limited, with many unable to afford maintenance inhalers. Similarly, in high-risk populations eligible for lung cancer screening, the incorporation of spirometry into community-based lung health checks proved both feasible and revealing. In a study from Manchester, nearly half of those with airflow obstruction did not have a prior COPD diagnosis, and more than half of these undiagnosed cases were symptomatic. Detection of undiagnosed airflow obstruction was more likely in men, younger individuals, those with lower smoking exposure, and even among those who were asymptomatic. These findings underscore the urgent need to prioritize early diagnosis and screening through both targeted community programs and integration with existing health initiatives to bridge gaps in COPD care and improve outcomes, especially where the burden of disease is highest [108]. Access to essential interventions, including patient education, vaccinations, pulmonary rehabilitation, and smoking cessation support, remains extremely limited, with only a small fraction of diagnosed patients able to obtain even short-acting bronchodilators. Maintenance treatments, such as inhalers, are often unaffordable, with the cost of just 30 days of therapy exceeding a low-skilled worker’s average daily wage. These challenges are compounded by guidelines that are often not adapted to the unique risk factors and phenotypes prevalent in LMICs, as well as substantial health system barriers to diagnostic and therapeutic access [9]. Addressing these unmet needs through improved case detection, context-specific care pathways, and better access to affordable interventions is crucial to reducing the burden of COPD in the world’s most affected populations.

Overall, the diagnostic pathway for NS-COPD in LMICs is hindered by a combination of atypical clinical manifestations, overlapping symptoms with other respiratory diseases, low provider awareness, and substantial resource constraints. Addressing these challenges will require concerted efforts to increase recognition of non-smoking risk factors, expand access to appropriate diagnostic tools, and develop context-specific guidelines tailored to the realities of LMIC healthcare systems.

## 5. Treatment Constraints

Non-smoking COPD patients in LMICs encounter substantial barriers to effective treatment, with the most prominent challenges arising from poor medication availability, high out-of-pocket costs, and limited access to diagnostic tools [9,12]. Many essential inhaled medications are frequently unavailable in healthcare facilities, and when present, the cost of maintenance inhalers can exceed a low-skilled worker’s daily wage, making long-term therapy largely unaffordable for most patients [109]. Consequently, adherence to prescribed inhalers is extremely poor, with over 80% of patients demonstrating inadequate adherence. The predominant reasons for poor adherence are financial hardship, as well as frequent erratic and deliberate non-adherent behaviors driven by the inability to afford consistent medication supplies [109]. In one LMIC population, more than two-thirds of patients specifically cited financial barriers as the main obstacle to proper adherence. These findings highlight the urgent need for systemic interventions to improve medication availability and affordability, as well as patient support strategies to enhance adherence in resource-constrained environments [109].

Access to essential COPD medications in low-income countries is severely limited. Most people with COPD in these settings cannot obtain or afford recommended inhaled therapies, leading to high rates of underdiagnosis, undertreatment, and preventable harm. Inhaled medications (such as short-acting beta-agonists (SABAs), inhaled corticosteroids (ICSs), and combination inhalers) are often unavailable in public and private pharmacies in many low- and middle-income countries (LMICs) [110]. Only a small fraction of countries meet the WHO target of 80% availability for essential COPD medicines. For example, only six of 58 countries met this target for SABAs, and none for ICS-LABA combinations [110,111]. When available, cost remains a critical barrier: SABA inhalers can cost 1–4 days’ wages, ICS 2–7 days, and ICS-LABA combinations at least 6 days’ wages for the lowest-paid workers. In some settings, the only affordable COPD medications are oral treatments, despite these not being recommended for long-term management [109,110,111].

Most COPD cases remain undiagnosed and untreated, with up to 95% of COPD cases identified through screening in LMICs never having been previously diagnosed, and only 4–6% of diagnosed cases receiving the recommended therapies. Poor medication availability and high out-of-pocket costs lead to very low adherence rates; over two-thirds of patients report financial barriers as the primary obstacle to consistent medication use [112]. Maintenance inhalers, crucial for long-term COPD management, remain especially scarce and unaffordable, even for patients with severe disease [113].

The systemic challenges include weak healthcare systems, inadequate supply chains, the absence or poor implementation of local guidelines, and limited awareness among both healthcare providers and the public. Essential medicines are often listed on national formularies but are not effectively stocked or distributed in practice, resulting in persistent shortages [10].

Access to COPD medications in low-income countries is critically inadequate due to poor availability and high costs, particularly for inhaled therapies. This results in widespread underdiagnosis, undertreatment, and preventable suffering. Addressing these gaps necessitates coordinated efforts to strengthen healthcare infrastructures, improve medication supply chains, reduce treatment costs, and enhance awareness across all healthcare system levels.

Diagnostic limitations further complicate management, as a lack of access to spirometry and other diagnostic resources leads to most COPD cases remaining undiagnosed until advanced stages [109,111]. Additionally, under-resourced healthcare systems and a shortage of respiratory specialists mean that COPD care is often delivered by primary care providers without specific training in respiratory disease management [111].

Non-pharmacological interventions are essential in the management of COPD, particularly for non-smoking patients in LMICs. These interventions, including pulmonary rehabilitation, increased physical activity, nutritional management, patient education, vaccination, and comprehensive care models, have been shown to significantly improve health-related quality of life (HRQoL), reduce symptoms, prevent exacerbations, and decrease hospitalizations, complementing pharmacological treatments [114,115,116,117]. Pulmonary rehabilitation, which integrates exercise training, education, and psychosocial support, is especially effective across age groups and disease severities, while regular physical activity and individualized exercise programs further contribute to reduced dyspnea and improved function. Nutritional support, such as screening for undernutrition and oral supplementation, is particularly important for older adults and can lower the risk of hospital readmission [115,117,118]. Education and self-management programs empower patients to better understand and manage their disease, leading to improved outcomes and treatment adherence [118]. Vaccinations against influenza and pneumococcus are strongly recommended to decrease the risk of exacerbations and mortality [115]. Additional approaches, such as non-invasive ventilation for selected patients and multidisciplinary care models, including tele-monitoring, further optimize management and compliance [119]. Despite clear benefits and cost-effectiveness, non-pharmacological interventions are often underutilized, particularly in LMICs, due to limited access, lack of resources, and low awareness among both patients and providers. Addressing these barriers and adapting interventions to local contexts and patient needs are essential steps toward optimizing COPD care and improving outcomes, especially for non-smoking patients in resource-constrained settings [118,120].

## 6. COPD Disparities: LMICs Versus Developed Countries

COPD represents a major global health concern, with notable disparities in prevalence, clinical management, access to healthcare, diagnostic resources, and mortality between LMICs and developed (high-income) countries. Over 80% of global COPD cases and approximately 90% of related deaths occur in LMICs, driven by heightened exposure to risk factors such as tobacco smoking, household air pollution from solid fuels, poverty, and limited healthcare infrastructure [2,111]. In these regions, COPD prevalence is markedly higher, exacerbated by late diagnosis, widespread underdiagnosis reported at rates of up to 95% in certain studies, and inadequate healthcare resources [9]. Conversely, developed countries generally report lower COPD prevalence, attributed to reduced exposure to traditional risk factors, better public health infrastructure, and improved early detection programs [121].

Recent analyses from the Global Burden of Disease (GBD) forecasting project highlight the critical role of tobacco control policies in shaping COPD outcomes globally. Forecast scenarios suggest that substantial health gains can be achieved through accelerated tobacco control policies aimed at reducing smoking prevalence, particularly in LMICs. The study emphasizes that maintaining existing tobacco policies alone will not suffice to significantly reduce smoking-attributable burden; instead, aggressive new policies are essential to achieving marked improvements in life expectancy and reducing years of life lost (YLLs) due to COPD and other smoking-related diseases. The modeling projects a potential gain of up to 1.5 years in global life expectancy among males if smoking prevalence were eliminated immediately, illustrating the profound impact of tobacco control policies on public health [122].

Clinical management and diagnostic capabilities differ substantially between LMICs and developed countries. In LMICs, healthcare systems face significant constraints, including severe shortages of critical diagnostic tools such as spirometry, inadequately trained healthcare personnel, and limited availability of essential COPD medicines, particularly inhaled corticosteroids and bronchodilators [12]. Essential medications for COPD treatment, particularly inhalers, frequently remain unavailable or unaffordable, costing patients the equivalent of one to seven days’ wages, substantially impacting adherence and effective disease control [123]. In contrast, developed nations typically enjoy broader and more equitable access to diagnostics, specialist respiratory care, comprehensive pulmonary rehabilitation programs, and affordable pharmacological treatments, facilitating timely interventions and better disease outcomes.

The divergence in healthcare access and guideline implementation further amplifies these disparities. National COPD guidelines in LMICs are either lacking or inadequately tailored to local contexts, often resulting in suboptimal care, low guideline adherence, and limited preventive measures such as vaccination programs. Conversely, developed countries maintain robust and contextually relevant guidelines, significantly enhancing patient outcomes through targeted and evidence-based management strategies [121].

Overall, addressing the significant COPD burden in LMICs requires multifaceted interventions, including enhancing respiratory care capacity, scaling up human resource training, expanding diagnostic availability, improving access to affordable essential medications, and adopting globally coordinated efforts for optimized COPD management. Strengthening healthcare infrastructure and formulating locally adapted, comprehensive clinical guidelines represent critical pathways towards reducing global disparities in COPD prevalence, clinical management, and mortality [111].

## 7. Public-Health and Policy Strategies

**Figure 3:**
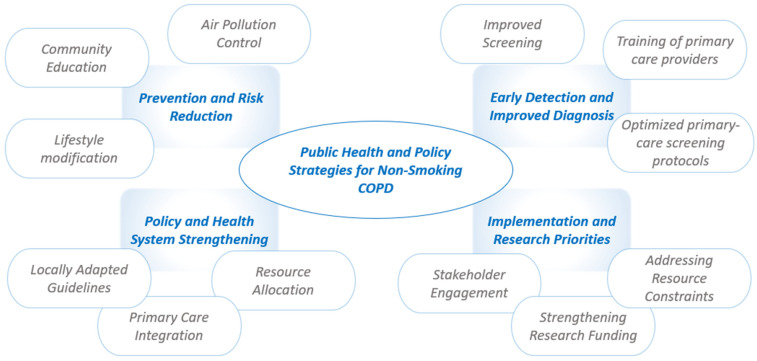
Public health and policy strategies for non-smoking COPD in low- and middle-income countries (LMICs).

Public health and policy strategies addressing non-smoking COPD in LMICs must target unique local risk factors, resource limitations, and implementation challenges. While tobacco remains a major cause of COPD globally, non-smoking COPD in LMICs is often linked to indoor and outdoor air pollution, occupational exposures, malnutrition, and early-life health issues (Figure 3).

Effective prevention approaches include improving household air quality through cleaner stoves and alternative fuels, enforcing occupational safety measures, and implementing robust vaccination programs for influenza and pneumococcus [121]. Policy actions by governments, such as regulating air quality, promoting clean energy solutions, and integrating COPD prevention within broader non-communicable disease (NCD) frameworks are essential [124]. However, many LMICs lack comprehensive COPD guidelines tailored to their local contexts, making effective implementation difficult. Challenges are compounded by limited access to diagnostic tools such as spirometry, an inadequate availability of medications and rehabilitation programs, and under-resourced health systems with insufficiently trained primary care providers [121]. In such settings, primary care practitioners and community health workers play essential roles in COPD management, delivering health promotion, screening, risk-factor reduction interventions, and supporting adherence. Improved screening and early diagnosis of COPD in LMICs require the development and implementation of affordable, accessible diagnostic tools specifically adapted for primary care settings. To facilitate earlier disease detection and effective management, it is essential to establish specialized training programs for primary healthcare providers and community health workers [10]. Additionally, prioritizing research into cost-effective prevention and pulmonary rehabilitation strategies, expanding primary care-based screening capabilities, and understanding context-specific risk factors are urgently needed to address the growing burden of non-smoking COPD in resource-limited settings [10,12]. Despite the current reliance on spirometry and clinical features, the search for reliable blood-based biomarkers in COPD remains ongoing. Recent studies have highlighted the potential role of surfactant protein B (SP-B) as a prognostic biomarker. In a prospective cohort, plasma SP-B levels were found to be significantly higher in patients with COPD compared to controls, and an increase in SP-B over 12 months was correlated with a greater decline in FEV1, suggesting its utility in monitoring disease progression. However, further research is needed to confirm its reliability and to clarify its role in clinical practice [125].

Key prevention efforts should prioritize reducing exposure to both indoor biomass smoke and ambient pollutants through government-led initiatives and community-based education programs aimed at raising awareness of these non-smoking risk factors. Moreover, strengthening primary healthcare infrastructure and ensuring availability and affordability of essential COPD medicines and pulmonary rehabilitation services is critical. Successful implementation also depends on stakeholder engagement, context-sensitive approaches, and sustained investments in COPD-focused research, particularly to address the limited resources and infrastructure challenges common in LMICs [10,12].

Vaccination is a key preventive strategy for patients with COPD, who face an increased risk of infection and related complications. Recommended vaccines, including those for influenza, pneumococcal, pertussis, RSV, SARS-CoV-2, and varicella zoster, significantly reduce exacerbations, hospitalizations, and mortality in patients with COPD. Influenza vaccination notably decreases influenza-related acute respiratory illness (ARI) and exacerbations, with vaccine effectiveness ranging between 76% to 85%, though it does not prevent non-influenza ARIs [126,127,128,129]. Pneumococcal vaccines effectively lower the risk of community-acquired pneumonia (CAP) (number needed to treat, 21) and COPD exacerbations (number needed to treat, 8), particularly benefiting patients under 65 or those with severe airflow obstruction [130,131]. However, vaccine efficacy can vary due to impaired immune responses associated with COPD, aging, and comorbidities, and live attenuated vaccines may not offer additional advantages over inactivated vaccines [126,132]. Despite these benefits, vaccination uptake remains suboptimal, and immune response variability necessitates ongoing research to optimize vaccine strategies. Recent advances in pneumococcal conjugate and RSV vaccines promise further reductions in infection risks and COPD exacerbations [130,131].

To effectively address the unique challenges faced by LMICs, COPD guidelines should prioritize non-smoking risk factors, such as household air pollution from biomass fuels and occupational hazards, incorporating community education and strategies for climate resilience, including the management of temperature extremes and indoor humidity [1,133]. Diagnostic criteria must be simplified, emphasizing clinical assessment based on symptoms such as cough, sputum production, and dyspnea, especially in settings lacking spirometry, as studies have validated the accuracy of this approach [10]. Cost-effective tools, such as peak flow meters, should be adopted to replace expensive diagnostic equipment. Treatment protocols require practical modifications using nebulized bronchodilators, short courses of oral corticosteroids, and promoting home-based pulmonary rehabilitation exercises in lieu of resource-intensive, center-based programs [120]. Ensuring access to essential medications listed by the WHO, including affordable options such as theophylline and short-course prednisolone, is crucial, and innovative solutions such as creating inhaler spacers from recycled bottles can significantly enhance drug delivery. Implementation strategies should include task-shifting to train community health workers, thus reducing reliance on specialists, and leveraging the existing program infrastructure for medication distribution and patient monitoring [133]. Prevention efforts must focus on clean cookstove initiatives to lower particulate exposure and targeted use of cost-effective vaccines. These adaptations are essential for closing critical care gaps while remaining aligned with global standards.

International and national organizations must commit to developing LMIC-specific guidelines, ensuring medication accessibility, and investing in training and research to effectively address COPD’s growing burden in these regions [134]. Collectively, these strategies can significantly reduce the COPD burden in LMICs, emphasizing the need for comprehensive and locally relevant interventions beyond traditional tobacco control measures.

## 8. Future Research

Future research should prioritize high-quality, longitudinal studies employing standardized diagnostic criteria and objective exposure assessments to strengthen causal associations between non-smoking risk factors and COPD in LMICs. There is an urgent need to identify specific biomarkers and genetic or epigenetic factors associated with COPD susceptibility and progression in populations exposed to biomass smoke and other environmental pollutants. Implementation science research is essential to determine effective strategies for integrating affordable diagnostics and treatments into primary care systems in resource-limited settings. Furthermore, investigations into culturally adapted, community-based pulmonary rehabilitation and educational interventions are critical for improving patient outcomes and adherence to management plans. Multisectoral collaboration to develop LMIC-specific guidelines and preventive policies, incorporating insights from local stakeholders and affected communities, represents another key area for targeted efforts.

In parallel, public health and policy initiatives must prioritize targeted interventions to address non-smoking risk factors, such as the widespread implementation of clean cooking technologies and improved ventilation to reduce household air pollution, stringent regulation and monitoring of ambient air quality, and the enforcement of occupational health standards to minimize workplace exposures. Enhanced investment in primary healthcare infrastructure is vital to ensure greater access to diagnostic tools such as spirometry and affordable medications, while integrating COPD prevention and management into broader non-communicable disease programs will facilitate community-based screening, early diagnosis, and patient education. Strengthening vaccination programs, fostering culturally tailored pulmonary rehabilitation and adherence support systems, as well as promoting international collaboration, capacity building, and sustainable financing models are essential to reduce COPD-related morbidity and mortality in LMICs.

## 9. Limitations

This review has several limitations that warrant consideration. First, the heterogeneity of studies conducted in LMICs, including variations in diagnostic criteria, exposure assessments, and outcomes measured, makes direct comparisons challenging. Many studies have relied heavily on symptom-based diagnoses or indirect exposure proxies rather than objective, standardized measurements, potentially affecting the accuracy of the findings. Additionally, this review predominantly addresses studies conducted in selected geographic regions, which may limit their generalizability to other LMICs with distinct environmental and socioeconomic contexts. The scarcity of longitudinal data and randomized controlled trials specific to NS-COPD phenotypes further constrains definitive conclusions regarding causality, disease progression, and optimal management strategies.

## 10. Conclusions

Non-smoking COPD in LMICs is driven by diverse and interconnected factors, including household and ambient air pollution, occupational exposure, early life insults, chronic infections, and socioeconomic disparities. These non-traditional risk factors result in distinct clinical phenotypes, presenting unique diagnostic and therapeutic challenges due to atypical presentations, limited resources, and inadequate healthcare infrastructure. Comprehensive public health and policy strategies, tailored guidelines, and strengthened primary care capacities are urgently required to mitigate this growing burden of disease. Effective intervention will rely on increasing awareness among healthcare providers, improving access to diagnostics and affordable medications, expanding non-pharmacological interventions, and adapting guidelines to local conditions. Ultimately, addressing these issues through coordinated global and local efforts will significantly enhance respiratory health outcomes in affected populations.

## Data Availability

The data presented in this study are available upon request from the corresponding author.
